# Research on the pathophysiology, treatment, and prevention of suicide: practical and ethical issues

**DOI:** 10.1186/s12888-019-2301-6

**Published:** 2019-11-01

**Authors:** Allison C. Nugent, Elizabeth D. Ballard, Lawrence T. Park, Carlos A. Zarate

**Affiliations:** 1 Section on the Neurobiology and Treatment of Mood Disorders, National Institute of Mental Health, National Institutes of Health, Bethesda, MD USA; 2 Magnetoencephalography Core Facility, National Institute of Mental Health, National Institutes of Health, Bethesda, MD, USA

**Keywords:** Suicide, Bioethics, Depression, Suicidal ideation, Research

## Abstract

**Background:**

Despite decades of research, the rate of death from suicide is rising in the United States. Suicide is a complex and multifactorial phenomenon and, to date, no validated biomarkers that predict suicidal behavior have been identified. Only one FDA-approved drug to prevent suicide exists, and it is approved only for patients with schizophrenia. Although anti-suicide psychotherapeutic techniques exist, treatment takes time, and only preliminary data exist for rapid-acting therapies.

**Discussion:**

While more research into suicidal ideation and acute suicidal behavior is clearly needed, this research is fraught with both practical and ethical concerns. As a result, many investigators and bioethicists have called for restrictions on the types of research that individuals with suicidal behavior can participate in, despite the fact that the available empirical evidence suggests that this research can be done safely. This manuscript presents background information on the phenomenology of suicide, discusses the current state of treatment and prevention strategies, and reviews the practical and ethical issues surrounding suicide research in the context of available empirical data.

**Summary:**

Currently, the causes of suicide are poorly understood, in part due to the fact that very few studies have investigated the acute suicidal crisis. Although some biomarkers for predicting risk have been developed, none have been sufficiently validated. The most successful current interventions involve means restriction. However, while numerous hurdles face researchers, these are not insurmountable. The available evidence suggests that research into suicide can be conducted both safely and ethically.

## Background

In 2018, the Centers for Disease Control announced that the suicide rate in the United States (US) had risen 30% between 2000 and 2016, reaching a rate of 13.5 per 100,000 [1]. They estimated that the suicide rate had increased 1% per year from 2000 to 2006 and 2% per year from 2006 to 2016. This increase in suicide rates occurred across the US, regardless of geography, and appeared to be particularly driven by the increased suicide rate of middle-aged women [1, 2]. Other research has linked these data to current public health crises for both alcohol and opioid deaths [3, 4]. Notably, if these suicide rates continue at current trends, an estimated 54,000 individuals per year will die by suicide in the US by 2025 [3]. Furthermore, this increase is occurring at a time when mortality rates from heart disease, cancer, stroke, and chronic respiratory disease are decreasing [5].

Worldwide, suicide is the second leading cause of death for 15–29 year-olds [6]. In 2012, approximately 75% of global suicides occurred in low- and middle-income countries [6]; as an example, in 2015, 34 farmers and agricultural laborers in India took their lives every day [7]. Nevertheless, suicide does not only result from economic distress. In South Korea, for example, the suicide rate was a staggering 32 per 100,000 in 2015 [8]—the highest rate in the developed world—despite the fact that South Korea had the 11th highest gross domestic product (GDP) in 2016 [9]. These sobering statistics underscore the complex and multifactorial nature of suicide and serve as a bleak reminder that no country or culture is immune. This manuscript lays out an ethical justification for expanding research into the pathophysiology, treatment, and prevention of suicide.

At the outset, we wish to first constrain the scope of our discussion. In particular, we will not comment on the issue of physician-assisted suicide or voluntary euthanasia in the context of intractable or terminal medical illness. We will also not comment on recent conversations regarding the extension of physician-assisted euthanasia programs for patients with psychiatric disorders who are not terminally ill [10–12]. This manuscript is limited to exploring those suicides that involve a person consciously deciding to take his or her own life without the aid of others, outside of any regulatory framework, and in the absence of any terminal illness. Although there is philosophical debate over the general ethical permissibility of suicide on the grounds of personal autonomy and libertarian ideals (for a review, see [13]), we are operating from the premise that suicide (as defined above) should, in general, be prevented wherever possible.

It is also useful at this point to comment on nomenclature [14]. Suicidal ideation is the presence of thoughts regarding suicide. These range from the passive (e.g., “I’d be better off dead”), to considering a specific suicide method, to developing specific suicidal intent with plans to act (for a definition of each of these concepts, please see assessments such as the Columbia Suicide Severity Rating Scale [15]). Suicidal ideation may be transient or chronic, with some individuals experiencing ongoing passive suicidal ideation for years without making any attempt. However, blanket terms such as “suicidal,” which are often used in the psychiatric literature, may not capture these nuances. Terms such as “active suicide crisis” are usually meant to indicate that an individual is experiencing more than just passive suicidal ideation; often, it means that an individual has intent to act on these thoughts or has even initiated a suicide attempt. These patients are often seen in emergency psychiatric settings and referred for immediate assessment and treatment. Finally, although research into the phenomenon of suicide has often involved those with a history of suicide attempt rather than active suicidal ideation or behavior, our use of the term “suicide research” specifically involves patients in either an acute crisis or with current ideation, unless otherwise indicated.

## Discussion

In order to set the context for our discussion, this paper will begin by introducing the multifactorial causes of suicide worldwide and then present a broad overview of current strategies for prevention and treatment. We will then discuss how research into suicide has previously been conducted. Crucially, little research into the active suicidal crisis has been conducted, leaving clinicians with few tools to treat acute ideation or behavior. The remainder of the manuscript discusses practical and ethical issues surrounding research with participants with active suicidal ideation or recent suicidal behavior. This includes issues of consent, issues particular to experimental therapeutics trials, the myth of the iatrogenic potential of suicide research, and regulatory and policy related concerns. Throughout, we explore current discussions in the bioethical literature and synthesize results from empirical research that have relevant ethical implications for research on suicide. Our overarching conclusion is that there is a moral imperative to conduct suicide research, and that this research can be performed safely and ethically.

### Causes of suicide

In 1897, Emile Durkheim authored a groundbreaking treatise of the phenomenon of suicide from a socio-cultural perspective [16]. Durkheim conceptualized suicide as stemming from four different factors encompassing ideas of community integration, sacrifice, moral confusion, and desperation [16]. Although suicides certainly occur for these reasons, his model failed to predict the trends in suicide deaths seen in modern times and may also not adequately capture the contributions of mental illness [17]. In this context, a retrospective study of suicide-related emergency room visits found that 82.7% of presenting patients had a concurrent mental disorder, most commonly mood disorders, substance or alcohol related disorders, and anxiety disorders [18].

Although psychological theories exist about the underlying causes of suicide (see, for instance, the review by Klonsky and colleagues [19]), most current thinking from the neurobiological literature on suicide characterizes self-harm as occurring from a stress-diathesis model, whereby life stressors precipitate a suicidal crisis in individuals with a pre-existing diathesis that encompasses aggressive and impulsive personality traits as well as pessimism [20–22]. In addition, authors such as Klonsky and May have argued for a three-step theory, with the causes of suicidal ideation rooted in pain and hopelessness and with social connectedness serving as a third—and protective— factor against the escalation of ideation to behavior [23]. Models such as this and others [24] decouple the processes behind ideation and behavior, as first suggested by Joiner [25].

Although suicide has largely been medicalized in the research literature, societal and cultural aspects undoubtedly contribute to suicide rates. This is especially evident when examining minority groups that may suffer from discrimination. For example, suicide rates are higher in those who identify as lesbian, gay, bisexual, or transgender (LGBT), likely due at least in part to hostility and/or marginalization of this community [26]. Another example is that of indigenous populations. For instance, the suicide rate for American Indian/Alaskan Native (AI/AN) individuals between 2007 and 2009 was 18.5 per 100,000, 1.6 times greater than the all-race suicide rate in the US of 11.6 for 2008 [27]. The disparity is particularly striking for males in the 15–24-year old group, where AI/AN suicides occur at a rate of 58.7 per 100,000; in comparison, the all-race US suicide rate for males of the same age group is 16.0 per 100,000.

Geopolitical factors may also contribute to suicide rates. For example, the highly publicized cluster of 12 suicides by Chinese workers at the Foxconn factory (which made Apple iPod and iPad devices, among other electronic products) were thought to be primarily attributable to employee abuse, unethical labor practices, and failure to admit culpability [28]. Worldwide, economic desperation is recognized as a frequent cause of suicide. The 2009 global financial crisis may have accounted for 5000 suicide deaths, and the US economic downturn in 2007/2008 is estimated to have increased suicide deaths in those with low education levels by 1.22 deaths per 100,000 [29]. Evidence also suggests that the strict austerity measures in Greece and other European nations during this time, along with rising unemployment, contributed to an increase in suicide rates [30].

Marked differences in suicide rates also exist across different countries. It should be noted that attitudes towards suicide continue to vary across cultures and religions, and reporting may therefore be more or less accurate depending on geopolitical region. According to the World Health Organization (WHO), the global average suicide rate was 10.7 per 100,000 in 2015 [8]. The Eastern Mediterranean region, encompassing the Middle East and Northern Africa, had the lowest reported rate at 3.8 per 100,000, while Europe had the highest rate at 14.1 per 100,000 [8]. A study examining differences in suicide rates across European nations found that both economic and climatic variables had significant effects [31]. Another relevant factor may be cultural attitudes towards suicide. For instance, a recent study found that Chinese psychiatrists showed more stigmatizing attitudes and less empathy towards individuals with mental illness than non-physician urban community members [32]. Importantly, evidence suggests that stigma against suicide may increase suicide risk in vulnerable individuals [33].

It should be clear at this point that the etiology of suicide is complex and multifactorial. While many suicides occur in the context of mental illness, other social, cultural, economic, and even political factors are frequently involved. Thus, it is clear that prevention strategies will likely need to address multiple domains outside the medical model of suicide.

### Treatment and prevention

Currently, no validated biological markers and few demographic or behavioral markers exist that predict suicide with high specificity and sensitivity [22]. In this context, the most potent predictor of future suicidal behavior is past suicidal behavior [21]. Repeated suicidal behavior is highly prevalent; in one study of 28,700 children, adolescents, and young adults in Ireland, 19.2% of patients engaged in another act of self-harm in the first year following the initial incident [34]. Although the precise genetic factors underlying a tendency towards self-harm are unknown, around 50% of the risk for suicidal behavior appears to be heritable [22]. Predicting and preventing suicidal behavior is fortunately a growing field of research, and several potential candidate markers have been identified for further study. One recent investigation evaluated a large array of biomarkers, then verified the most promising candidates in an independent sample. Apolipoprotein E (ApoE) and interleukin-6 (IL-6) emerged as markers, potentially indicating the involvement of inflammation and accelerated aging [35]. Potential epigenetic and genetic markers of suicide also exist, although gene expression interacts with life events, and further replication is needed [36]. Numerous studies have also identified altered sleep architecture as a biomarker of suicidal thoughts and behavior [37–39].

In general, prevention strategies can be divided into two categories: a “high-risk” approach that targets individuals at high risk, and a “population” approach that targets social and environmental factors [40]. Zalsman and colleagues [41] recently published a comprehensive overview of both risk-based and population-based suicide prevention strategies studied over the past 10 years. Strikingly, they concluded that the most robust evidence supported practical population-based measures, such as reducing access to drugs, toxins, and jumping sites. Although research into possible links between accessibility to firearms and suicide risk has been very limited in the US, the CDC nevertheless reported that, in 2017, 60% of deaths by firearms were suicides and only 37% were homicides [42]. Research into restricting the means of suicide—and potential interactions with cultural factors—can be difficult, and research assessing the actual impact of policy changes is even more so. In addition to restricting access to the means of suicide, adequate treatment of psychiatric disorders—either through pharmacological means or psychotherapy—has also been shown to reduce suicide rates [41]. The study by Zalsman and colleagues also found sufficient evidence to support the implementation of school-based education programs, but noted that despite individual positive studies on primary care screening programs, gatekeeper training, and telephone or internet interventions, the evidence remains inadequate to support large-scale deployment [41]. Nevertheless, it is important to acknowledge that despite the use of current evidence-based prevention strategies, suicide rates, at least in the US, have increased rather than declined [1]; it should also be noted here that the strategies discussed above are, in general, primary prevention strategies.

As noted above, laws, regulations, and structural changes can also alter suicide rates. For instance, in the former Soviet Union, an anti-alcohol campaign initiated by former president Mikhail Gorbachev between 1985 and 1988 strikingly reduced the number of suicide deaths; following the collapse of the Soviet Union in 1991, rates began to sharply increase again [43]. Another, and perhaps more surprising, example is that suicide rates by gas inhalation in the United Kingdom decreased dramatically as the percentage of carbon monoxide in domestic gas (used for home heating and cooking) decreased between 1955 and 1975, as utilities transitioned from coal gas to natural gas [44]. Surprisingly, although non-gas inhalation suicides increased somewhat in younger men, overall suicide rates decreased substantially, again emphasizing that removing easily accessible means of attempting suicide can be an effective prevention method.

In addition to the dearth of prevention strategies, few evidence-based medical practices exist to treat suicide risk. In terms of medications, the antipsychotic clozapine is the only FDA-approved drug for the treatment of suicide risk, but it is specific to patients with schizophrenia. Evidence also exists that the mood stabilizer lithium [45] and the N-methyl-D-aspartate (NMDA) modulator and rapid-acting antidepressant ketamine [46, 47] may exert anti-suicidality effects, along with electroconvulsive therapy (ECT) [48]. Indeed, ketamine has been used off-label in the clinic to treat active suicidal ideation or behavior, and the use of esketamine (recently approved by the FDA for treatment-resistant depression in adults) [49] may not be far behind, although there are relatively few prospective trials. A recent meta-analysis of studies that examined ketamine as an agent for reducing suicidal ideation found evidence to support its use in the clinic but emphasized the need for further randomized, controlled trials of adequate power [50]. Psychotherapeutic interventions have also been investigated, with evidence supporting the efficacy of cognitive behavioral therapy and dialectical behavioral therapy [51–53] in addition to other suicide-targeted therapies [54]. It should be noted that these treatment strategies could be considered both secondary or tertiary prevention strategies, designed to prevent both relapse and recurrence of suicidal ideation or behavior.

Any discussion of suicide treatment and prevention would be incomplete without addressing access to care. According to the WHO, low-and middle-income countries have fewer than 0.5 psychiatrists per 100,000 people [55]. In the US in 2011, there was an average of 10.9 psychiatrists per 100,000 people, though access varied substantially by region [56]. Financial access to care is also an issue. In 2010, 16% of US citizens lacked health insurance, although that number had declined to 9.1% by 2015, in large part due to government programs designed to increase access to care [57]. Nevertheless, one study found that 76.9% of individuals who attempted suicide had contacted a health care provider within the last 3 months, potentially indicating missed opportunities for prevention [58]. Transitional access is also important; while an emergency room visit or brief psychiatric hospitalization can forestall a death due to suicide, without further transition plans to community care, patients are at risk for future attempts. In fact, the weeks after discharge from psychiatric hospitalization are the period of highest risk for suicide [59, 60].

Presently, suicide risk is difficult to assess with specificity and sensitivity, although numerous biomarkers are being pursued. In addition, while treatment strategies exist, their effectiveness is not always well established, and population-level interventions may have the greatest impact, at least on overall suicide rates. Although a full discussion of this topic is well beyond the scope of this manuscript, the existence of better screening and prevention methods introduces an ethical question regarding the appropriate degree of paternalism that could be exerted in an effort to reduce suicide rates. This is related to the idea of quaternary prevention, whereby prevention strategies should be formulated in a manner that prevents undue overmedicalization of a population [61]. While mandatory screening and treatment may lower suicide rates in the future, this may be an unacceptable intrusion on the rights and liberties of private citizens.

### Current research into suicidal behavior

The statistics cited above suggest that current research into suicidal behavior is inadequate. The last five decades of research have had no tangible impact on suicide rates in the US overall; in fact, suicide rates have increased. Although policy and practical approaches can reduce the number of suicides, these measures do not directly address the underlying causes. However, basic science investigations are hampered by the fact that no animal model for suicide currently exists, although work is underway to develop such models [62]. In addition, while post-mortem studies have revealed numerous isolated abnormalities, no overarching mechanism has been identified. Replication also remains a problem, as does the extremely limited number of post-mortem brains available for research [63].

As noted above, little research exists regarding the treatment of an active suicidal crisis. Most neurobiological suicide studies typically compare individuals who have a history of suicide attempt with individuals who have no such history, such that the observation time point is far removed (in many cases, by years) from any neurobiological or behavioral changes taking place at the time of the attempt (for a review of neuroimaging studies, see [22]). While this research may be able to reveal trait-type differences between individuals, very little can be concluded about what precipitates a suicidal crisis from a biological perspective or how it can be treated. In addition, most studies investigate suicide only in the context of a mental illness, usually major depressive disorder, bipolar disorder, or schizophrenia.

### Practical and ethical issues surrounding research into suicidal behavior

#### Practical issues

While it should be clear at this point that more research into the phenomenon of suicide is needed, the reasons behind the lack of progress are multifactorial. From a practical standpoint alone (ethical issues will be discussed later), research into the active suicidal crisis is, quite simply, difficult. Access to research participants and recruitment are significant issues [64], as one cannot simply place an ad in the newspaper for individuals who want to end their own lives. Adequate recruitment generally requires partnering with an emergency department or psychiatric inpatient unit (for example, see [65]). Aside from the logistical difficulties, the population in any given local emergency room may differ from typical research samples in terms of education level, socioeconomic status, and medical comorbidities. In addition, individuals experiencing an active suicidal crisis may be medically unstable, particularly if a drug overdose was attempted.

Another key issue is that in order to be safe for an actively suicidal population, a research environment may have to be modified to remove items that could be used for self-harm, in line with procedures effectively used at clinical inpatient facilities [66]. Additional staffing may also be needed to adequately supervise patients. In a litigious society, researchers may be reluctant to study patients at risk for suicide, fearing that they will be liable for any attempted suicides that occur during research; certainly, clinicians may be unsure that their practices protect them from legal liability in the case of patient suicide [67]. Although none of these issues is insurmountable, they represent barriers that may be difficult to overcome, particularly in highly competitive funding environments. However, it should be noted that, given the potentially fluctuating course of suicidal ideation, research on patients with past suicidal ideation but no recent or current suicidal behavior may nevertheless require the same additional safety measures, again raising the costs and complexity of such research. Finally, institutional review boards (IRBs) may be reluctant to approve research involving active suicidal ideation or behavior [64].

#### Ethical issues: consent

In this brief discussion of issues related to consent in suicide research, we specifically refrain from discussing suicide research in subjects who overtly lack the capacity to provide informed consent, regardless of whether they are suicidal or not, as in most cases this research would be decidedly unethical [68]. We would also like to emphasize that we are specifically discussing consent to research participation, in contrast to consent/assent for clinical care. Throughout this section, we maintain that no psychiatric patient who researchers suspect may be significantly harmed by enrolling in research should be allowed to participate, regardless of their wishes. In this case, the principle of non-maleficence should—and frequently is—used to trump personal autonomy. Furthermore, it is reasonable to assert that the point at which non-maleficence can overrule patient autonomy may be more conservative for the actively suicidal participant than for a subject with only passive ideation. For the purposes of this manuscript, we are not discussing patients who are involuntarily committed because of their suicidal ideation and/or behavior.

One common hurdle to enrolling actively suicidal individuals in research surrounds the validity of informed consent [69]. Intuitively, we can speculate that if an individual wishes to end his or her own life, their ability to adequately appreciate the risk to benefit ratio of a research study is questionable. We can also question the authenticity of such decisions, and how much a suicidal patient’s decision may be clouded by their illness [70]. Mishara and colleagues [13] noted that it is our “social instinct” to classify “the vast majority of suicidal individuals as vulnerable”. It is unclear whether it is appropriate or useful to classify those with suicidal ideation as “vulnerable”, at least in a regulatory sense; for a discussion of this issue in severe mood disorders, see Nugent and colleagues [71]. Presumably, a patient with suicidal ideation who voluntarily enters a clinic or research facility actively seeking treatment and/or research enrollment would rather see their suicidal ideation removed through treatment than through death. Subjects who have just attempted suicide who request treatment or enroll in a research study likely have the same motivations. Relatedly, although at least one report has suggested that suicide researchers may not have the same interest in preventing suicide as do suicide prevention workers [13], we believe this to be an overly pessimistic assessment. Indeed, it is our opinion that most researchers in the field of suicidality are highly and specifically motivated by a desire to prevent future suicides.

It is also important to point out that the threshold for competency to consent is somewhat fluid, depending upon the consequences of consent or refusal. For example, from a practical perspective the capacity required to *refuse* treatment is generally higher than the threshold required for capacity to consent to treatment. While capacity to consent to any research necessarily requires the presence of basic capacities (the ability to express a choice, understand facts, reason with those facts, and appreciate facts in context [72]), the nature of the research must be considered, particularly as it pertains to potential benefit and to the availability of other treatments. There may thus be a lower threshold for consenting to a trial offering potential benefit—especially in light of the few existing evidence-based treatments for an active suicidal crisis—than the threshold required to refuse treatment altogether. As long as informed consent is valid, it is further unclear from an ethical standpoint why a patient experiencing suicidal ideation who wishes to enroll or not enroll in a study should be treated differently than a non-suicidal research subject; this is particularly true given that suicidal ideation tends to fluctuate with time, so that in the span of a few weeks, ideation may have disappeared in one research participant and appeared in the other [73]. Indeed, some have suggested that classifying suicidal intent as a dichotomous variable—that is, as either present or not present—does not accurately reflect the nuance and subtlety of patients’ motivations [74]. Furthermore, it is unclear that classifying individuals as “suicidal” based either upon scores on standard instruments or clinical judgement accurately predicts future actions [69]. In sum, a blanket definition for a vulnerable population is no more appropriate for a suicidal population than for a mentally ill population. And although some suicidal individuals are competent to consent to research, safeguards such as routine consent monitoring and capacity assessment will allow these individuals to be identified and facilitate their enrollment.

Although we have discussed issues surrounding informed consent in subjects with severe mood disorders elsewhere [71], we know of no empirical evidence to support denying suicidal patients access to research. The only available data involve potential research subjects with psychiatric disorders in general. These studies have consistently shown that such patients generally possess the capacity to provide informed consent [75] and, furthermore, that they perceive mental health research as ethically acceptable, even for those experiencing “a lot of emotional pain” [76, 77]. Additionally, depressed patients overwhelmingly report altruistic motives for enrolling in research [77, 78]. Although the studies cited above did not specifically address whether or not they included participants with suicidal ideation, it is unclear how important the presence or absence of suicidal ideation is in the phenomenology of depression. In fact, the overall severity of depression is not a good predictor of suicidal behavior [79]. It is well recognized that individual symptoms of psychiatric disorders vary across cultures [80–82] and, as a result, it is conceivable that suicidal thoughts may be expressed only by patients who believe that self-harm is an appropriate manifestation of depression or emotional distress in the larger context of their ethnic/cultural/religious background.

#### Ethical issues in experimental therapeutics research

Provided that informed consent is valid, what kinds of trials should permit the enrollment of individuals at risk for suicide? In this context, it should be noted that suicidal individuals are commonly excluded from randomized controlled trials of new therapies [83]. Mishara and Weisstub [13, 83] stated that including such subjects in placebo-controlled clinical trials is “generally considered unethical”, an assessment echoed by Stanley [84]. A consensus statement from many prominent psychiatric researchers similarly stated that trials for those at significant suicide risk should not be placebo-controlled [14], even while noting that the common practice of excluding patients at risk for suicide is hard to justify. Unfortunately, in part due to lack of research, it is difficult to quantify “significant risk” for suicidal behavior. Furthermore, non-suicidal depressed patients can and do become suicidal depressed patients. That is, regardless of whether subjects were suicidal or not at screening, suicidal ideation tends to fluctuate with time [85]. As a result, a subject who is not suicidal at enrollment may nevertheless become so weeks or even days later, or vice versa [86, 87]. Real-world examples of this are underscored by several key studies. One meta-analysis incorporating data from 19,639 depressed patients enrolled in antidepressant trials found that the incidence of suicide attempts (*n* = 130) and completed suicides (*n* = 19) did not differ between those randomized to receive the investigational drug, an active comparator, or placebo [88]. A follow-up study of 48,277 participants came to similar conclusions [89]. Another study examining clinical bipolar disorder research studies found no completed suicides or suicide attempts in 11 placebo-controlled trials for an acute manic episode; in contrast, the researchers found two suicides and eight suicide attempts in four placebo-controlled trials to prevent manic or depressive episodes, all of which occurred in the active compound group [90]. Another retrospective study found that emergent suicidal ideation during clinical trials was short-lived and not exacerbated by research procedures [91]. Perhaps most importantly, in 24 pediatric trials of selective serotonin reuptake inhibitors (SSRIs), which carry a black box warning of suicide risk, there were no deaths from suicide in 4582 patients [92]. Thus, the practice of excluding research participants with suicidal ideation does not prevent suicidal ideation and behavior during clinical trials; rather, it bolsters the dearth of knowledge about how to treat individuals at risk for suicide by decreasing generalizability.

It should be clear from the studies described above that excluding research participants with suicidal ideation does not prevent suicidal behavior in participants in clinical trials. Thus, the larger point embedded in this discussion is that although every effort should be made to eliminate suicides occurring in the context of research, this precaution should be undertaken *regardless* of whether or not an enrolled subject was suicidal at enrollment or at some other arbitrary time point, given the potentially fluctuating course of suicidal ideation. Clinical trials of at-risk patients should include periodic suicide risk assessments, clear procedures for participants to contact clinical staff if their suicidal thoughts worsen over the course of the study, and comprehensive safety plans to implement in the event of a suicide crisis, created jointly by patients and clinical staff in advance of study participation [93]. Conversations about potential suicide risk assessment and response that occur before research begins also make IRB and/or other regulatory agencies aware that suicidal thoughts and behaviors are not unexpected outcomes when working with patients with mental illnesses. In addition, clear procedures for removing a patient from research and/or beginning new treatments due to suicidal thoughts/behaviors should be clearly delineated to study staff so that they do not have to improvise such procedures during a clinical crisis [64].

Another commonly made argument against including suicidal patients in research studies is that delaying treatment to suicidal patients is unethical. Yet, as reviewed above, most current FDA-approved treatments have no demonstrated effect on suicidal behavior. Therefore, most patients who seek treatment for suicidal thoughts/behaviors may already be undertreated or treated in an experimental fashion as a matter of course, and this should be considered when determining the risk/benefit ratio for any research protocol. In addition, SSRIs, which are the most common FDA-approved antidepressants, may take weeks to exert an antidepressant effect, and months before the full response is realized [94]. In contrast, investigational agents (such as ketamine) have the potential to trigger a more rapid antidepressant response [95].

We believe that most clinical trials should consider including suicidal individuals; again, we reiterate the caveats that consent must be valid, patients must not be at imminent risk of harm, and all necessary safety monitoring procedures must be provided. Inpatient placebo-controlled trials of rapid-acting agents, which may be short in duration, may be particularly amenable to including suicidal individuals [96]. While researchers and ethicists may state that “evidence-based, severity-based interventions should be available” [14], the sad fact is that no evidence-based, robustly effective, and widely available treatments for acute suicide risk exist. Given the points argued above, we disagree with the notion that psychiatric research and the protection of life are mutually exclusive. Although research with suicidal participants may require more personnel, more advance preparation, and more frequent contact (or inpatient hospitalization), we believe that this research can be successfully conducted while nevertheless respecting the safety and overall welfare of patients. As noted previously, given the lack of reliable markers for the onset of suicidal ideation or suicidal behavior, anyone enrolled in a psychiatric research trial may become suicidal. Including any level of suicidal ideation as a criterion for immediate withdrawal may have the paradoxical effect of reducing safety by encouraging subjects experiencing ideation to hide this fact from research staff. Furthermore, this practice does nothing to increase the safety of suicidal patients overall.

#### The myth of the iatrogenic potential of suicide research

A final area where empirical data may be useful is the widespread fear that discussing suicide may precipitate suicidal thoughts in research participants. A 2015 study of human research ethics committee members revealed substantial barriers to approving research studies involving suicidal participants [64]. In that survey, 65% of respondents expressed concern that suicide risk might be exacerbated or reinforced by suicide research, in part due to requiring subjects to recall or revisit their experiences. However, the available data overwhelmingly demonstrate the exact opposite: discussing suicide with patients often reduces distress and suicidal thoughts [97–100]. In addition, the majority (17/20) of a group of patients with bipolar disorder enrolled in a study that discussed suicide stated that they did not find talking about suicide to be distressing, and 15/20 viewed the research as valuable and worthwhile [101]. Another study found that using images or words relating to self-injurious behavior did not appreciably impact research participants’ desire to die [102]. Despite the ample evidence to the contrary, one commentator even suggested that because one cannot exclude the possibility of just one research subject attempting suicide after being asked questions about suicidal ideation, ethics committees should evaluate proposed research studies with this possibility in mind [103]. This misconception is not limited to ethics committees; a survey of 28 suicide researchers demonstrated widespread concern that research participation in general might exacerbate suicidality. In fact, data from our studies show the exact opposite [91]. Clearly, the continued perpetuation of the iatrogenic potential of suicide risk has hampered suicide research, particularly approval by ethics committees.

#### Suicide research: implications for policy and regulation

Given the reluctance of investigators to conduct research studies in suicidal individuals, we are left with a situation where a condition is treated with drugs or other therapies that have not been fully tested for that condition. As Fisher and colleagues state, “[m] ortality rates for suicidal individuals will not decrease if these individuals continue to be treated with inadequate and unproven interventions” [69]. Likewise, Pearson and colleagues [104] point out that, “[i] ndividuals at high risk for suicidality deserve to receive safe, well-tested effective treatments.” Another important point is that a significant proportion of patients suffering from depression are treatment-resistant [105], meaning that they do not respond (or respond only partially) to currently approved therapies. Thus, if a potential subject who has not responded to an adequate course of all FDA-approved therapies wishes to enroll in an experimental treatment study but has active suicidal ideation, they have very few options outside of research. If all the necessary safeguards are present, and if the research participant is competent to give informed consent, there would seem to be an obligation under the principle of justice to include them. Indeed, when considering other potentially fatal illnesses with no FDA-approved treatments, patients are generally not excluded from research because they are “too ill”. For example, patients with terminal cancer are regularly asked to participate in Phase I drug trials for which there is no expectation of benefit [106]. Protection of these subjects is ensured by robust informed consent procedures and careful monitoring, and the fact that most research participants die is not considered a failure of research—in fact, these trials are considered crucial to developing new ways to treat cancer [106]. It has even been proposed that suicide deaths in the context of research should not be considered unthinkable or even unexpected; after all, close to 800,000 people die by suicide every year outside of research, in part due to the lack of effective treatments [69]. Although researchers studying suicide should of course attempt to use any and all appropriate safeguards to prevent deaths, the fact that deaths may occur should not make research unacceptable, any more than deaths occurring in the context of cancer or infectious disease research. Research with suicidal participants should be viewed through the same lens that we use to research other fatal diseases.

Another critical point is that, given issues with access to care, it is not difficult to see why individuals with suicidal ideation might seek out research rather than treatment-as-usual in their community. In the United States, the average stay in a hospital following admission for a suicide attempt is 5.6 days [107], far less than the time required for an antidepressant to begin exerting effects, and far less than patients generally spend enrolled in a typical research study. Even a placebo-controlled study may be viewed as a reasonable option by an individual with suicidal ideation, given the thoroughness of the expected medical work-up, the intensity of contact with staff, the expectation of top-notch medical care, and the prospect of receiving standard care following the end of the research interval—again, provided that all necessary monitoring and rescue conditions are in place [108]. In fact, some authors have suggested that research may be viewed as in the best interests of even youth at increased risk of suicide, an arguably even more vulnerable population [109].

Despite even the most carefully implemented safeguards, any psychiatric subject may experience an active suicidal crisis during research, and subjects already suicidal at the time of enrollment may worsen during research and require involuntary commitment. As stated earlier, a discussion of research on involuntarily committed patients is outside the scope of this manuscript. Thus, it is sufficient to state here that these participants would be removed from research. One potentially important issue, however, is that in cases where emergent involuntary commitment of a research participant is necessary, issues arise regarding potential clinically necessary breaches of confidentiality [84]. However, the threat of imminent harm to the patients themselves or to others would necessitate breaching confidentiality, whether in research or in clinical practice. The fact that these breaches are more likely to occur if the population studied is explicitly suicidal is, we believe, an inadequate reason to prevent pursuing much needed research. In practice, consent forms should explicitly state under what conditions confidentiality would be breached in case of threats to safety.

Another ethical issue raised by Stanley [84] is the potential to uncover new issues that may arise from success in research into the phenomenon of suicide. For instance, suppose a biomarker for suicide risk is discovered—who should be screened for this risk? What rights and liberties can or should be violated in the interest of suicide prevention? How should persons possessing a suicide marker be informed, particularly if they deny any suicidal ideation? What are the ramifications for hiring, insurance coverage, life insurance claims, etc.? Finally, what is the ethical response if a biomarker is found but no successful treatment exists? While we accept that these issues are valid concerns, none of them render suicide research unethical. In fact, there are parallels to be found in the moral analysis of research into biomarkers for dementia [110]. Briefly, these issues include when to test, when to inform, and what actions to take. While more research may result in more issues to navigate, such issues are common to a vast array of disorders, and advancing the science of these disorders is the only way to advance the search for effective treatments and cures.

## Conclusions

Suicide continues to be a significant problem in the US and worldwide. For decades, clinicians and researchers alike have bemoaned the lack of reliable ways to predict and treat suicidal behavior as well as the dearth of research into the biology and psychosocial aspects of suicide [111, 112]. In addition, despite the research that has been performed and the numerous professional societies and scientific journals devoted to the problem, suicide rates have not fallen. While vast sums of money have been devoted to the study of the other major causes of death—for instance, heart disease, cancer, Alzheimer’s disease—the same is not true of suicide.

Given the enormous cost in terms of human life, it is our opinion that a moral imperative exists to support research into the causes and prevention of suicide. Furthermore, while we review many practical and ethical issues that complicate suicide research, it is our opinion that none of these issues is insurmountable. Although informed consent is often raised as an concern, we have presented evidence that consent can and should be considered valid in many cases. While some bioethicists have stated that participants with suicidal ideation should not be enrolled in placebo-controlled clinical trials, evidence exists that research does not exacerbate suicidal ideation and, furthermore, that excluding participants who may have few treatment options may deprive them of potential benefit. We also presented evidence that researchers and ethics committee members believe that research into suicidal ideation and behavior may worsen these symptoms, despite ample evidence demonstrating that this is simply not the case. Finally, although there may be future policy and regulatory issues if reliable biomarkers are discovered, this is not a reasonable justification to avoid research. In sum, ample empirical evidence supports the notion that research including individuals with suicidal ideation can be conducted safely. Indeed, regardless of any remaining issues, once we determine that an ethical obligation to study suicide exists, we must necessarily determine ways in which such research can be conducted ethically.

## Data Availability

Not applicable.

## Abbreviations

AI/AN: American Indian/Alaskan Native
ApoE: Apolipoprotein E
ECT: Electroconvulsive therapy
GDP: Gross domestic product
IL-6: Interleukin 6
IRB: Institutional review board
LGBT: Lesbian, gay, bixexual, or transgender
NMDA: N-methyl-D-aspartate
SSRI: Selective serotonin reuptake inhibitor
US: United States
WHO: World Health Organization
